# Defining and Evaluating Classification Algorithm for High-Dimensional Data Based on Latent Topics

**DOI:** 10.1371/journal.pone.0082119

**Published:** 2014-01-09

**Authors:** Le Luo, Li Li

**Affiliations:** Faculty of Computer and Information Science, Southwest University, Chongqing, China; Swiss Institute of Bioinformatics, Switzerland

## Abstract

Automatic text categorization is one of the key techniques in information retrieval and the data mining field. The classification is usually time-consuming when the training dataset is large and high-dimensional. Many methods have been proposed to solve this problem, but few can achieve satisfactory efficiency. In this paper, we present a method which combines the Latent Dirichlet Allocation (LDA) algorithm and the Support Vector Machine (SVM). LDA is first used to generate reduced dimensional representation of topics as feature in VSM. It is able to reduce features dramatically but keeps the necessary semantic information. The Support Vector Machine (SVM) is then employed to classify the data based on the generated features. We evaluate the algorithm on *20 Newsgroups* and *Reuters-21578* datasets, respectively. The experimental results show that the classification based on our proposed LDA+SVM model achieves high performance in terms of precision, recall and F1 measure. Further, it can achieve this within a much shorter time-frame. Our process improves greatly upon the previous work in this field and displays strong potential to achieve a streamlined classification process for a wide range of applications.

## Introduction

With the rise of the Web 2.0, social media such as Facebook and Twitter are not only popular, but are becoming a new way of life. At the beginning of 2012, Facebook has more than 800 million registered users worldwide, presenting huge amounts of user-generated content (UGC) which is accessible to the general public [1]. As a result, the world's data centers are now replete with exabytes of UGC due to UGC shares and uploads by individuals. The activities of a vast majority of internet users is limited to texts due to restraints of limited coverage and slow network traffic. The use of text categorization includes news classification, Web page classification, intelligent recommendation of personalized news, spam mail filtering, etc. Classifying texts fast and accurately, therefore, is becoming increasingly relevant in today's technology oriented world. However, large-scale texts always produce high-dimensional data, which pose the challenge of efficiently processing high-dimensional data while not affecting the quality of performance [2].

Traditional methods of text categorization are based on the Vector Space Model (VSM). In order to improve training speed and maintain the classification accuracy, many dimension reduction methods have been proposed [3]. These methods work to some extent. However, two common problems arise when dimension reduction is considered. Firstly, if the dimensionality is reduced to some threshold value beneficial to improving training speed, the classification accuracy will be compromised to an unsatisfactory level. Secondly, to keep the accuracy at a productive level, the dimensionality may reach over several thousands or even more if the original datasets are large. That is, it is hard to satisfactorily resolve the tradeoff between speed and accuracy of the algorithm. Both problems originate from performance of feature reduction methods which fails to recognize the relationship between words in the text and meaningful data lying within it.

To address these two problems, we present our method for classifying texts quickly and accurately. We utilize the Latent Dirichlet Allocation (LDA) algorithm [4] to generate features, equivalent to adding semantic information to VSM. We then employ the Support Vector Machine (SVM) on it. Through experiments on *Reuters-21578* (Reuters-21578 website. Available: http://www.daviddlewis.com/resources/testcollections/reuters21578/. Accessed 2013 Nov 3.) and *20 Newsgroup* datasets (20 Newsgroups website. Available: http://people.csail.mit.edu/jrennie/20Newsgroups/. Accessed 2013 Nov 3.), we found that using LDA as feature selection method can improve performance much more than other feature reduction methods. In other words, the combination of LDA and SVM outperforms all others in both classifier performance and training efficiency.

The remainder of the paper is organized as follows. Section 2 describes the problem. Section 3 discusses our approach using LDA+SVM. Section 4 evaluates the procedures experimentally followed by further discussion of relevant issues associated with models used in the paper. The related work is reviewed in Section 5. Finally Section 6 concludes the paper.

## Problem Description

### Problem Formation

Text categorization firstly requires formalizing the data collection so that it can be processed by classification algorithms, such as SVMs, KNN, and so forth. In the VSM, usually, a document is represented by a vector and thus the whole data collection is represented by a matrix, whose rows represent documents and columns represent terms. Details are provided as follows.

Constructing a VSM matrix is equivalent to constructing a document-term matrix, which maps each document and its extracted words into a vector. In this case, each vector represents a document and each of its elements corresponds to a word (or term) of a vocabulary extracted from all the documents. Suppose 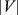 is the size of the vocabulary, that is, the number of different words extracted from the documents, the vector 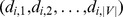 then represents the document 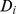, with the 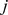-th element 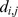 being a measure of the weight of the 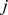-th term of the vocabulary in the document 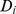. Among measures proposed for this weight, the Information Retrieval (IR) metric, called tf-idf, is adopted widely because of its good performance. In the VSM, the rows of the matrix represent documents, while the columns correspond to all the words from the vocabulary. The value of the elements is the weight of each word in the document.

### Dimension Reduction

In information retrieval, dimension reduction can be divided into feature selection and feature extraction. The former includes five methods: document frequency (DF), information gain (IG), mutual information (MI), a 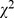 statistic (CHI), and term strength (TS). Yang [5] experimentally demonstrated that IG, DF and CHI have similar effects on the performance of the classifiers and perform better than the rest of the five. Moreover, DF method is algorithmically simple and thus it costs less time, which is superior in handling amounts of documents.

On the other hand, feature extraction transforms the data with high-dimensional features to the data with lower-dimensional features. The data transformation may be linear, such as principal component analysis (PCA), Latent Semantic Indexing (LSI) and Linear Discriminant Analysis, but many nonlinear dimensionality reduction techniques also exist, for instance, kernel PCA.

## New Approach for Text Classification

In this section, we propose the method of combination of LDA and SVMs. First, we illustrates how to employ LDA to generate the document-topic matrix—in which each row represents a document vector and each column represents topics a document vector contains, so topics are treated as features of document vectors (in following paragraph, when we say “topic features”, that refers to the topics of document vectors of the document-topic matrix), and then explained how to utilize the SVM to classify and predict the category of documents fast and precisely.

### Matrix of Topic Distribution

Latent Dirichlet Allocation (LDA) is a probabilistic model of a corpus that not only assigns high probability to members of the corpus, but also assigns high probability to other “similar” documents. The basic idea is that documents are represented as random mixtures over latent topics, where each topic is characterized by a distribution over words.

**Figure 1:**
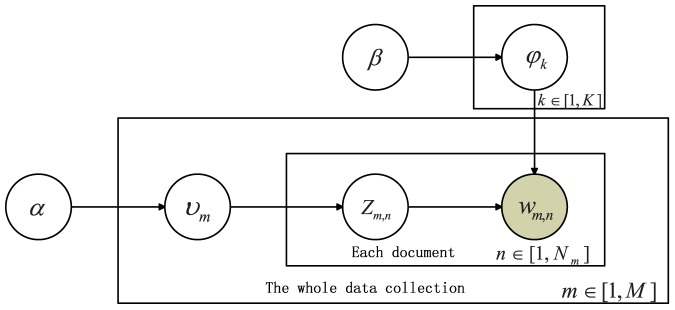
LDA: a generative graphical model.

As shown in Figure 1, a document 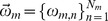 is generated by firstly picking a distribution over topics 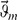 from a Dirichlet distribution 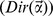, which determines topic assignment for words in that document. Then the topic assignment for each word placeholder 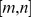 is performed by sampling a particular topic 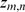 from multinomial distribution 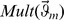. Finally, a particular word 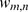 is generated for the word placeholder 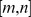 by sampling from multinomial distribution 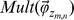, where Gibbs Sampling [6] is often adopted.

In the process, two matrices 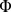 and 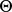 are computed as follows.
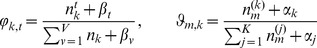
(1)The matrix 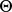 is just the matrix of topic distribution for documents, which is an important component of our method described in Section.

### Support Vector Machine (SVM)

SVMs are widely applied to many domains and yields better results than other learning algorithms, especially in classification. The basic principle of SVMs is described as follows:

Given a training set of instance-label pairs 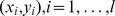, where 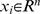 and 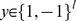, SVMs require the solution of the following optimization problem:
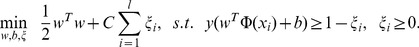
(2)Here training vectors 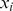 are mapped into a higher dimensional space by the function 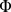. SVM finds a linear separating hyperplane with the maximal margin in this higher dimensional space. 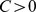 is the penalty parameter of the error instances. According to Mercer theorem [7], there always exists an equation 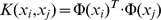 called the kernel function. Then after a series of derivations problem 2 can be rewritten as:
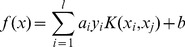
(3)Note that the number of dimensions to be dealt with turns from the number of dimensions of 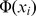 to that of 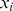. That's why SVMs can cope with the “curse of dimensionality” [8] and process high-dimensional data with good performance.

### Main Steps for Improvement

There are four steps in document classification in the context of dimension reduction. The proposed approach aims to retrieve optimal set of features to proceed towards lower time cost and higher performance. The optimal set of features should reflect the original data distribution.

1. building a document-term matrix according to the VSM;
2. analyzing the topic distribution and forming a matrix about topic distribution for documents;
3. utilizing the topic distribution values as the weight of VSM;
4. building the classifier to test the documents.

The topic analysis by LDA estimation with Gibbs sampling will generate two matrices 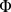 and 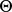. However, only matrix 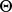 is useful to our study as it indicates the relationship between documents and topics. The matrix 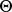 is used in Step 2. Step 4 uses SVM to build upon the characteristics identified in Step 2. Although the SVM converges slow in large dataset, it resolves the over-fitting and feature redundancy problem. This gives a great performance in terms of generalization. It thus yields better classification results than others. Using the topic features offsets the slow convergence resulted from SVMs. More detail will be presented in Section.

## Experimental Evaluation

To validate and gain insights about the usefulness of the proposed approach, we performed a set of experiments on classification on documents. Results are presented below followed by discussions.

### Datasets

To prepare the labeled training and test data for the experiments, we used the two large multi-class datasets *Reuters-21578* and *20 Newsgroups* to conduct our empirical study for text classification. Both datasets have been widely used in large scale text classification tasks, and are publicly available. Table 1 provides a brief description of them.

**Table 1:** Dataset Description.

Dataset	Category	Training data	Test data
Reuters-21578	acq	1519	691
	coffee	112	30
	crude	344	196
	earn	2700	1044
	grain	399	168
	interest	161	72
	money-fx	465	215
	ship	121	58
	sugar	97	43
	trade	298	142
20 Newsgroups	alt.atheism	480	319
	comp.graphics	584	389
	comp.os.ms-windows.misc	591	394
	comp.sys.ibm.pc.hardware	590	392
	comp.sys.mac.hardware	578	385
	comp.windows.x	593	395
	misc.forsale	585	390
	rec.autos	594	396
	rec.motorcycles	598	398
	rec.sport.baseball	597	397
	rec.sport.hockey	600	399
	sci.crypt	595	396
	sci.electronics	591	393
	sci.med	594	396
	sci.space	593	394
	soc.religion.christian	599	398
	talk.politics.guns	546	364
	talk.politics.mideast	564	376
	talk.politics.misc	465	310
	talk.religion.misc	377	251

**Figure 2:**
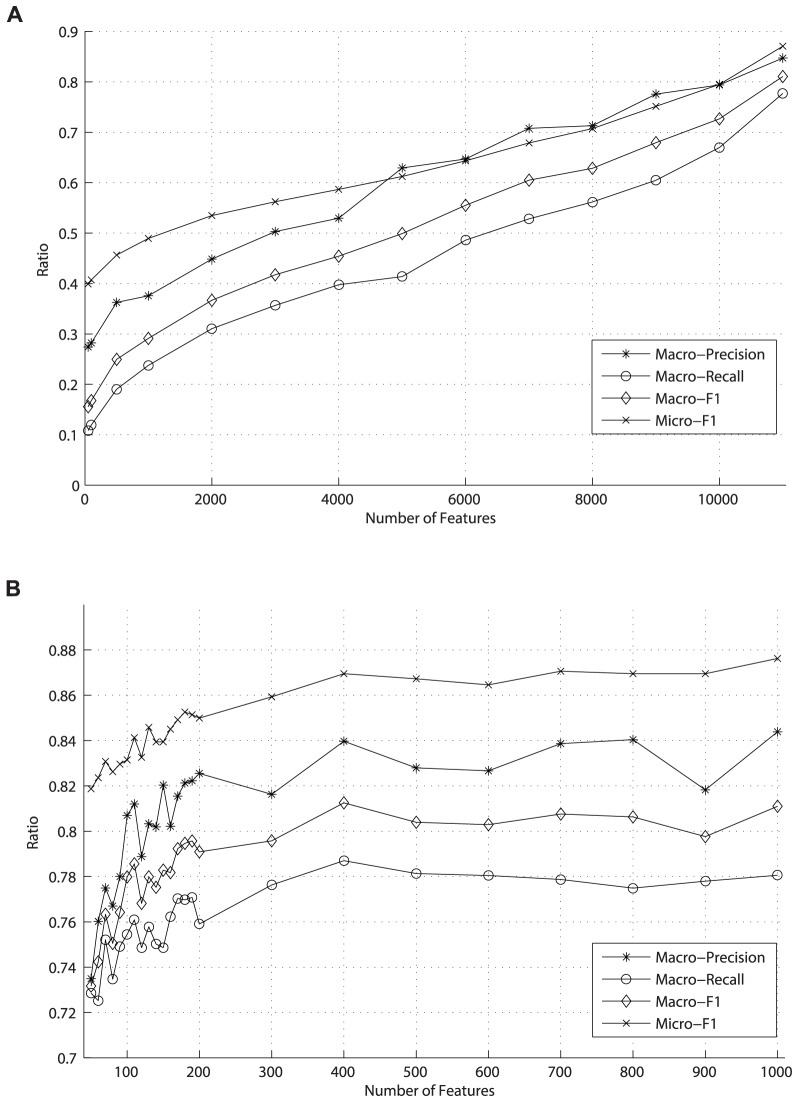
The values of Macro-Precision, Macro-Recall, Macro-F1 and Micro-F1 under different number of features reduced by DF method.

**Figure 3:**
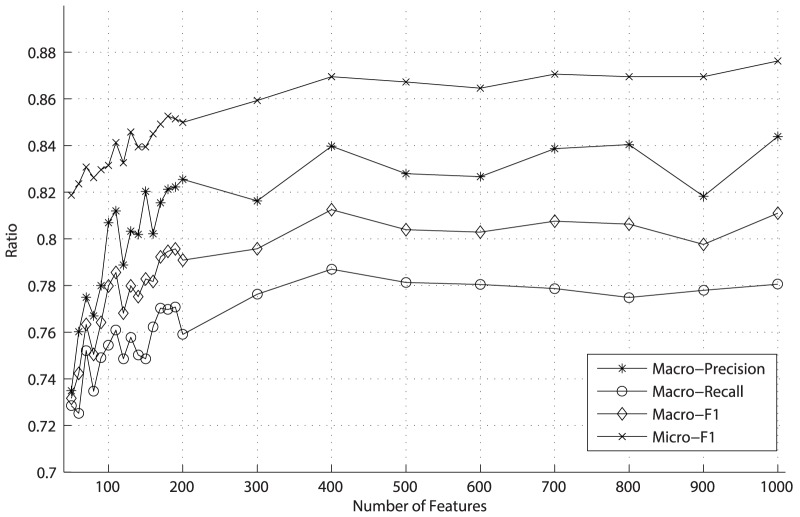
The values of Macro-Precision, Macro-Recall, Macro-F1 and Micro-F1 under different number of features reduced by PCA method.

**Figure 4:**
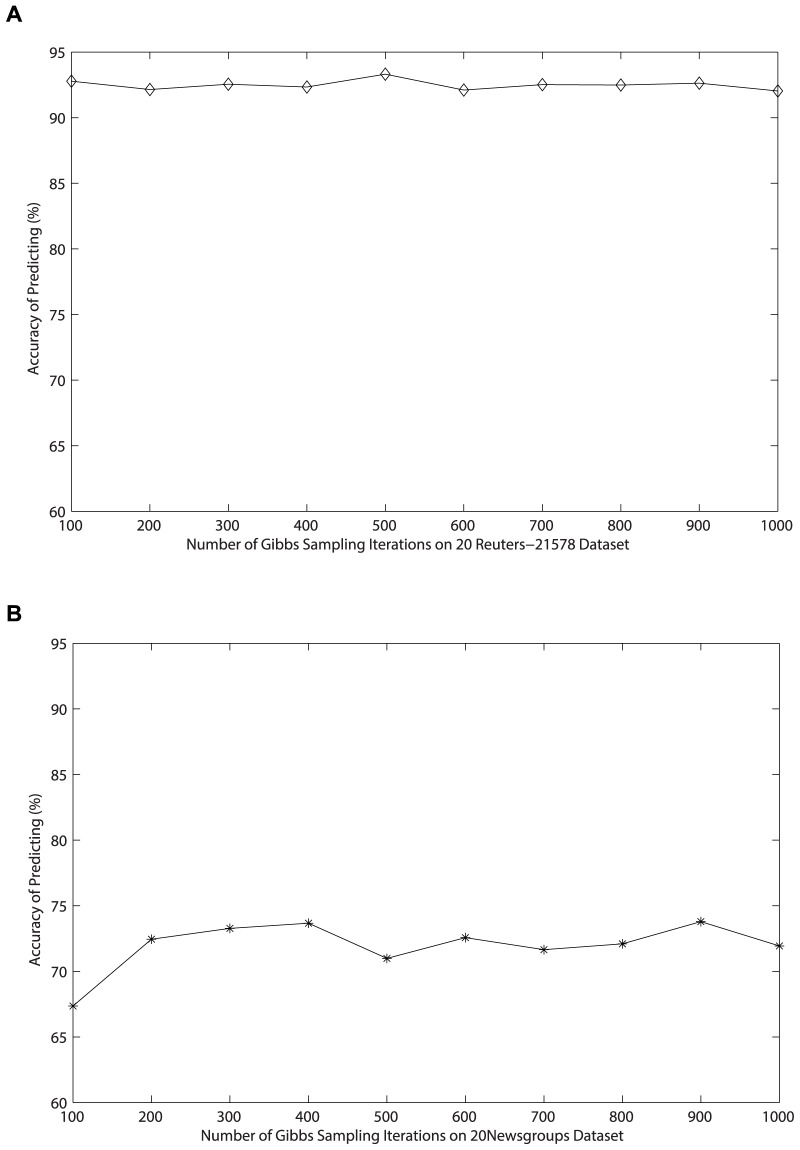
The values of Macro-Precision, Macro-Recall, Macro-F1 and Micro-F1 under different topic features from LDA method.

**Figure 5:**
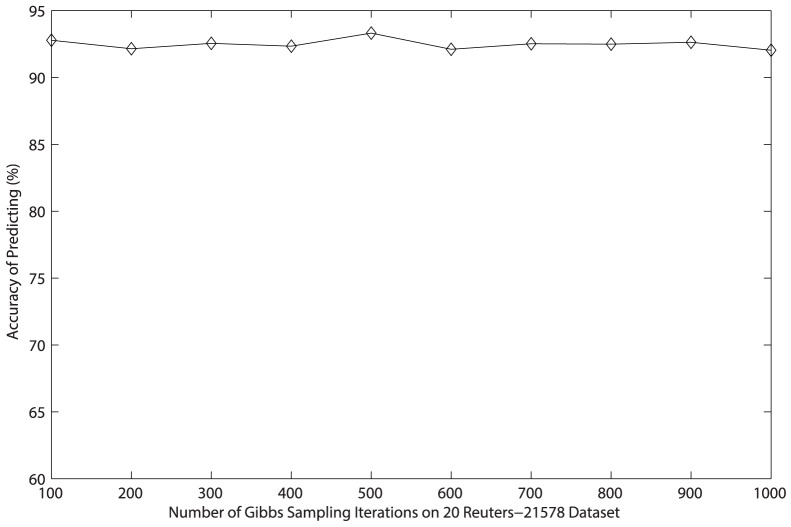
Relationship between accuracy and the number of Gibbs sampling iterations on *Reuters-21578* dataset.

**Figure 6:**
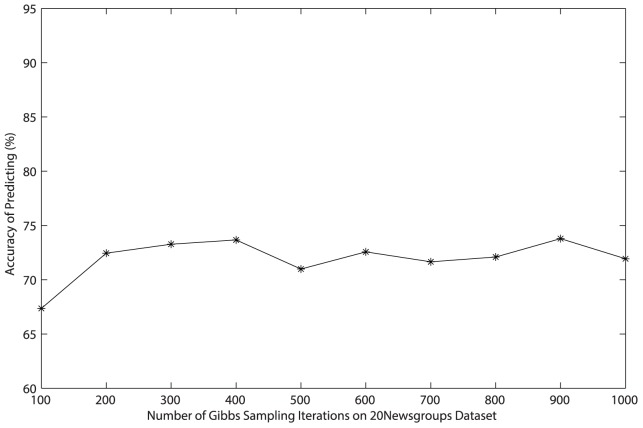
Relationship between accuracy and the number of Gibbs sampling iterations on *20 Newsgroups* dataset.

Initially, the datasets are not convenient for text categorization, and thus we preprocess them in the way of applying tokenization, stemming, punctuation and stop-word removal. After that, the data are ready to be processed by our method. Note that we only utilize dataset *Reuters-21578* to perform experiments with results depicted in Figure 2, Figure 3 and Figure 4, respectively. However, it is feasible to employ both datasets in the experiment described in Figure 5 and Figure 6 respectively under our experimental setting.

### Evaluation Criteria

In multiclassification, the 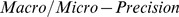, 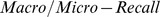 and 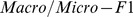 criteria are adopted to evaluate the classifiers. They are defined, respectively, as follows.
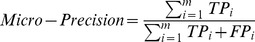
(4)

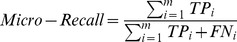
(5)

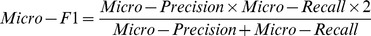
(6)

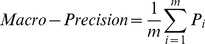
(7)

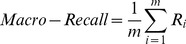
(8)

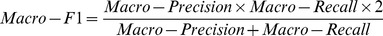
(9)


## Results and Analysis

For the purpose of comparison, we choose two dimension reduction methods, the DF and PCA, which are also selected as baselines. We firstly use these two methods to transform the documents into document-term matrices where the term is treated as features of a document vector. Then documents in the form of document vectors are trained and the category of new documents are predicted by the SVM where we adopt the SVM tool *LibSVM* (Libsvm tool website. Available: http://www.csie.ntu.edu.tw/~cjlin/libsvm/. Accessed 2013 Nov 3). The prediction accuracy is evaluated using Macro-Precision, Macro-Recall and Macro-F1, Micro-F1. Note that because each instance has exactly only one correct label, Micro-Precision and Micro-Recall are the same as Micro-F1. See Figure 2 and Figure 3.

Figure 2 and Figure 3 demonstrate the values of Macro-Precision, Macro-Recall, Macro-F1 and Micro-F1 under different number of features reduced by the DF and PCA method respectively. As shown in Figure 2, the values gradually increase as the numbers of features selected by DF method become larger. Obviously, the results are poor until the dimensionality reaches as high as 11000. That is, when DF method selects more than 11000 features, the classifier produced by the SVM performs well. In Figure 3, after the dimensionality is reduced by PCA to 400, the results of these evaluation measures begin to reach the burn-in period. Moreover, both dimension reduction methods can bring on good results of the classification measures, and the only difference between them is that the former leads to the best results by reducing the dimensionality to 11000 and the latter only to 400.

Different from the DF and PCA which treat terms as feature of document vectors, the LDA employs the topics as features of document vectors. We use the LDA tool *GibbsLDA++* (GibbsLDA++ website. Available: http://gibbslda.sourceforge.net. Accessed 2013 Nov 3.) to obtain the matrix of topic distribution for documents, namely, the document-topic matrix. Then a document can be treated as a VSM vector in which a topic is regarded as a feature whose weight is this topic's possibility distribution value—in this step, we utilize the *GibbsLDA++* to generate 10 to 200 topics document-topic matrix; for every 10 topics we conduct one trial. Finally, we adopt SVMs to conduct 5-fold cross validation and evaluate prediction precision, recall and F1 using the same measures as DF and PCA do. The results are shown in Figure 4.

As shown in Figure 4, when the number of topic generated by LDA reaches about 120, the Macro-Precision, Macro-Recall, Macro-F1 and Micro-F1 of classification fluctuate slightly around 0.87, 0.85, 0.86 and 0.94 respectively. In other words, using LDA to select only 120 features can lead to good classification results. Then a question arises: when will the curves decline? Theoretically, when the number of topics we obtain approaches the number of terms in the dataset, the curve will decline. Matrix 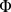, as described in Section, represents term distribution for topics, so if at one extreme case the number of topics is equal to that of terms, the topic features will not be different from term features the DF method selected.

Experimentally, we observed the results of the Macro-Precision, Macro-Recall, Macro-F1 and Micro-F1 are still about 0.87, 0.85, 0.86 and 0.94 respectively as expected when 1000 topics on *Reuters-21578* was chosen. Considering time consumed in the training phase, it is strongly recommended that dimensionality be reduced as much as possible. We tried different combinations and different numbers of feature dimension in our work. The LDA model provides valuable insight into dimension reduction and we finally got 120 dimensions. The experiment illustrated that the obtained features, though only about 120 dimensions, achieved better performance in all these criteria compared with DF and PCA methods. As such, we have achieved dramatic dimension reduction from original 11000 to the current 120, with nearly the same promising result.

Since it is not easy to control the convergence of Gibbs sampling when estimating parameters for LDA [6], the number of Gibbs sampling iterations we adopt is 1000 in foregoing experiments in order to insure the classification accuracy. To save time of the generation of the matrix of topic distribution, we need to estimate the number of iterations needed when the classification accuracy reaches the so-called “burn-in period”. In order to implement the experiment easily on two datasets, we generate only 50 topics as features and adopt the accuracy as the basic criterion. The number of Gibbs sampling iterations starts from 100 to 1000 where the step is 100, and results are shown in Figure 5 and Figure 6. Obviously, the accuracy obtained by experiments on *Reuters-21578* dataset keeps around 92% with 50 features as the number of iterations changing from 100 to 1000. The accuracy on *20 Newsgroups* dataset reaches “burn-in period” when the number of iterations exceeds 200. Obviously, cutting down the number of iterations is possible and necessary in order to achieve great efficiency.

To confirm the high performance of the feature selection method LDA, we conduct a comparison experiment using the baseline methods DF and PCA. In order to display and contrast the best performance these methods can reach, we take 11000 features selected by DF, 400 features extracted by PCA, and 120 features selected by LDA to perform the classification, and also classifiers are evaluated by the Macro-Precision, Macro-Recall, Macro-F1 and Micro-F1 measures. Results are displayed in Table 2.

**Table 2:** Classifier performance based on different feature reduction methods.

Method	Macro-Precision	Macro-Recall	Macro-F1	Micro-F1
DF+SVM	0.8471	0.7770	0.8105	0.8706
PCA+SVM	0.8397	0.7870	0.8125	0.8695
LDA+SVM	**0.8896**	**0.8554**	**0.8722**	**0.9361**

In Table 2, obviously, although the only 120 topics generated by LDA are used as features, the Macro-Precision, Macro-Recall, Macro-F1 and Micro-F1 measure values are better than that using DF and PCA method to reduce dimensions, both of which have used 11000 and 400 dimensions respectively but just get nearly the same results as LDA does. That is, LDA+SVM method uses only 120 features to perform classification and obtain better results than DF+SVM and PCA+SVM using 11000 and 400 features do.

Besides classification precision and recall, the LDA+SVM is also better than other two methods at training speed. The classification using the SVM, based on the DF method, needs to calculate the td-idf values as the weight to generate a document-term matrix. This is much more time-consuming than producing a matrix of topic distribution for documents. In our experiments, the setting is a 3.0 GHz processor and 2 G memory computer. With regard to the *20 Newsgroups* dataset containing 18846 files, it takes about 85 minutes to produce the matrix of topic distribution by *GibbsLDA++* with 100 topics and 1000 Gibbs Sampling iterations whereas about 240 minutes to generate a document-term matrix. Moreover, it takes about 120 minutes to employ PCA to extract 400-features matrix from *Reuter-21578* dataset containing 8875 files on the computer with 3.0 GHz processor and 4G memory (See Table 3). Therefore, the general classifications using SVMs based on the DF and PCA not only consumes more time in the process of training data by the SVM, but also in the process of preprocessing documents before training. Our approach hence surpasses the general classification model using SVMs in terms of precision, recall and efficiency.

**Table 3:** Time consumed by the three methods generating the training or input matrices.

Methods	File quantity	Time consumed	Experimental setting	Dimensionality generated
DF+SVM	18846	about 240 mins	3.0 GHz processor	11000
			2G memory	
PCA+SVM	8875	about 120 mins	3.0 GHz processor	400
			4G memory	
LDA+SVM	18846	85 mins	3.0 GHz processor	110
			2G memory	

Therefore, our proposed method of combination of LDA and SVM takes less time and achieve higher performance than the traditional dimension reduction and classification methods.

## Discussion

The proposed approach is promising in terms of classification efficiency. Below are further discussions which produce interesting insights into using different models in text classification problems.

The function of LDA lies in supplying semantic information to the VSM model. The performance of our method, fundamentally, is benefitted from LDA. It can find the latent topics from the whole dataset with topics represented by a distribution over words. Features selected by DF method with tf-idf values will miss the associated documents, even if these documents belong to the same class but they share different words. In contrast, it can associate documents belonging to different classes if these documents share same words. That is, the DF method fails to recognize the potentially valuable relations of polysemy and synonymy. In addition, the document-term matrix using to DF select features is sparse because it is impossible that each word in the vocabulary exists in one document. All of this leaves the DF+SVM method rather prone to classify documents into wrong classes. Although PCA method copes with the synonymy, the polysemy is still missing from it. The LDA method, instead, takes it into consideration and thus can overcome these shortcomings of the DF and PCA. LDA+SVM obtains best performance among these methods.

The SVM is a good choice for the LDA+SVM method. There are many learning methods, such as k nearest neighbors, Naive Bayes, maximum entropy, SVMs and so on. With the exception of the shortcoming of slow convergence, SVMs surpass all of them in dealing with over-fitting and feature redundancy problem and robustness and thus in classification accuracy [9] [10] [11]. To offset this shortcoming, we can approach to right reduction such as 120 dimensions used in the paper. The experiment illustrated that our approach performs very well in high-dimensional data classification.

### Related work

Topic model is a powerful and popular tool in machine learning and natural language processing. Most related to our method, Cai et al. [12] utilize topic features constructed by the LDA algorithm to improve the word sense disambiguation on unlabeled data: their method achieves an improvement over the simple naive Bayes classifier. Different from their method, we mainly adopt the SVM and LDA to boost the accuracy of document classification. Another similar work is also employing LDA to analyze the latent topics of documents by Phan et al. [13], but they mainly discover hidden topics from external large-scale data collections and use a semi-supervised learning method to process short and sparse documents. Besides, Tang et al. [14] [15] successfully utilize the topic model in expertise search and patent mining.

The VSM is an algebraic model for representing text documents. It treats a text document as BOW (bag of words), and its term weight is often tf-idf values. Besides the VSM, Nunzio et al. in [16] propose a bidimensional representation of textual documents for text categorization. However, methods proposed in these papers improve the accuracy only on some small-scale test datasets. Thus, the VSM is still the main way of text representation despite its neglecting semantic information among words and documents. Since the VSM is prevalent, dimension reduction has to be performed. There are lots of methods of dimension reduction such as BNS (bi-normal separation) [17], GI [18], LSI [19], etc. Anyway, dimension reduction will spend considerable time in text classification.

In addition to the SVM, there are some other models or methods being applied, such as the maximum entropy model [20], Bayes, kNN, etc. These methods are all restricted to the conventional classification model which is based on similarity measurement. Considering the lower accuracy with more classes in multi-classification, Hao et al. [21] present a novel binary hierarchical classification method that recursively decomposes a multi-class problem into several binary-class problems. This method can really enhance the accuracy, but it must build the classification models many times when the number of classes is large, which is very time-consuming.

## Conclusion

In our study of text classification, we found two prevalent problems which must be resolved in order to streamline the classification process. The first problem regards dealing with the high-dimensional nature of data and the second problem is maintaining high performance with only a subset of data. As discussed earlier, the challenge arises in finding the ideal tradeoff between the accuracy and efficiency, which is the focus in this paper.

In this study, we have presented the LDA+SVM method and achieved good results in text classification. The usage of LDA in the paper makes the best possible features with important semantics between terms. We have: (1) figured out how many dimensions or features to use when approximating the matrix; (2) significantly reduced the dimensionality of training data, boosting the training speed; (3) enhanced the classification accuracy. The experiments showed that our method of combining LDA with the SVM can achieve high performance using a combination of appropriate features of previous works. Our approach is competent in dealing with high-dimensional data of user-generated text content, which has wide ranging applications in industry and business.
